# The Long Gestation of the Small Naked Mole-Rat (*Heterocephalus glaber* RÜPPELL, 1842) Studied with Ultrasound Biomicroscopy and 3D-Ultrasonography

**DOI:** 10.1371/journal.pone.0017744

**Published:** 2011-03-07

**Authors:** Kathleen Roellig, Barbara Drews, Frank Goeritz, Thomas Bernd Hildebrandt

**Affiliations:** Leibniz Institute for Zoo and Wildlife Research, Berlin, Germany; University of Illinois at Chicago, United States of America

## Abstract

The naked mole-rat (*Heterocephalus glaber*) is one of the two known mammalian species that live in a eusocial population structure. Here we investigate the exceptionally long gestation period of 70 days observed in the mole-rat queen. The course of seven successful pregnancies in two individuals was recorded in a colony of captive naked mole-rats using ultrasound biomicroscopy (UBM) and 3D-ultrasonography. We establish a catalogue of basic reference ultrasound data for this species by describing the ultrasonographic appearance of reproductive organs, calculating growth curves to predict gestational age and defining ultrasonographic milestones to characterize pregnancy stages. Mean litter size was 10.9±2.7, of which 7.2±1.5 survived the weaning period. Mean interbirth interval was 128.8±63.0 days. The reproductive success in our colony did not differ from previously published data. In the queen the active corpora lutea had an anechoic, fluid filled centre. Using UBM, pregnancy could be detected 53 days before parturition. The period of embryonic development is assumed to last until 30 days before parturition. Embryonic resorptions were detected frequently in the queen, indicating that this might be an ordinary event in this species. We discuss the extraordinary long gestation period of this small rodent and postulate that the long gestation is beneficial to both the eusocial structure and longevity. An increased litter size, twice as large as for other rodents of similar size, seemingly compensates for the doubling of pregnancy length. We demonstrate that the lifetime reproductive effort of a naked mole-rat queen is equivalent to the mass of offspring that would be produced if all of the females of a colony would be reproducing.

## Introduction

The naked mole-rat (*Heterocephalus glaber*, Rüppell 1842) is one of the two known and studied mammals with an eusocial behavioural pattern [1], [2]. The classical definition of eusociality referred to the social organization of insects and includes the division of reproductive labour, cooperative care of offspring and overlap of generations [3]. It was recently postulated that eusociality evoled as a result of standard natural selection rather than of selection of kin and encompasses a series of defined stages [4]. In addition to the two well studied species, the naked and the Damaraland mole-rats (*Cryptomys damarensis*, Ogilby 1838), other members of the Bathyergidae (*Cryptomys spp*.) family also display cooperative breeding behaviour (a single female and a small number of males reproducing at any one time within a colony) [5]. According to the original definition of eusociality the reproductive caste of an individual is a life-long status and lifelong residency of individuals within their natal colony is required, since both are not necessarily fulfilled in the case of the naked mole-rat it was discussed whether the social structure of these animals can clearly be defined as eusocial [6], [7], [8].

Despite these argumentations, the reproductive biology of naked mole-rats displays some unique features among mammals. Usually within a colony only a single female and one to three male individuals are actively involved in reproduction. Wild colonies can comprise as many as 300 individuals but still only one queen would reproduce. In some captive colonies it was found that two females reproduce, however this only happened during unstable transient periods [9]. Furthermore it was shown that females can become reproductively active as soon as eight days after removal from their natal colony [10]. The reproducing female, the queen, can be distinguished from the other females by her body length, which increases during pregnancy [11]. The ovaries of the queen are larger and its uterine horns are generally wider and thicker walled compared to non-breeding females [12]. The queen can deliver four to five litters per year. The observed mean interval between litters was 79.6 days ranging from 77 to 84 days [9] and postpartum oestrus and mating can occur after seven to eleven days [9], [10]. Hence, the gestation length was calculated to be 66 to 76 [9] and 72 to 77 days [10]. For placental mammals it was demonstrated that the length of gestation is positively correlated to the animal's body weight [13]. Hence it is astonishing that the small naked mole-rat with a body weight of about 40 to 50 grams, has such an extraordinary long pregnancy duration.

Even though behavioural suppression by the queen seems to play a dominant role [14] it is not yet fully understood, how the reproductive suppression of all the non-breeding individuals of a colony can occur so effectively. Studies investigating hormonal patterns indicated that the suppression of reproduction of non-breeding females was due to a failure of corpus luteum development probably resulting from a block of ovulation [15], [16]. In pregnant females progesterone levels were significantly elevated at the beginning of pregnancy (days 1 to 20), increased in mid gestation and dropped down just before parturition (days 61 to 70). Even when reproductively active females were not pregnant their progesterone levels were still elevated for prolonged periods, suggesting that ovulation and subsequent development of corpora lutea had occurred. In contrast progesterone levels in reproductively suppressed animals were undetectable at any time. The hormonal pattern in assumingly pregnant females supports the observation of a gestation length of about 70 days. Litter size is variable but usually comprises nine to ten pups with a range of one to 27 pups [9]. After the altricial offspring are born, they are nursed exclusively by the queen for about four weeks. Other colony members are involved in the care for the offspring, too. Compared to other mammals, this species can give birth and successfully nurse almost twice as much offspring as there are mammary glands on the queen [17].

Due to the many interesting aspects of the biology of this species like its unusual social structure and its longevity of more than 28 years [18] a lot of research was performed in the naked mole-rat which is accumulated for the interested reader [19], [20]. However, there are many aspects of reproductive physiology that need to be further elucidated. We conducted a study using several modes of high-resolution ultrasound technique to display embryonic development in the naked mole-rat to get more insight in the extraordinary long gestation period of 70 days.

## Materials and Methods

### Animals

**Figure 1:**
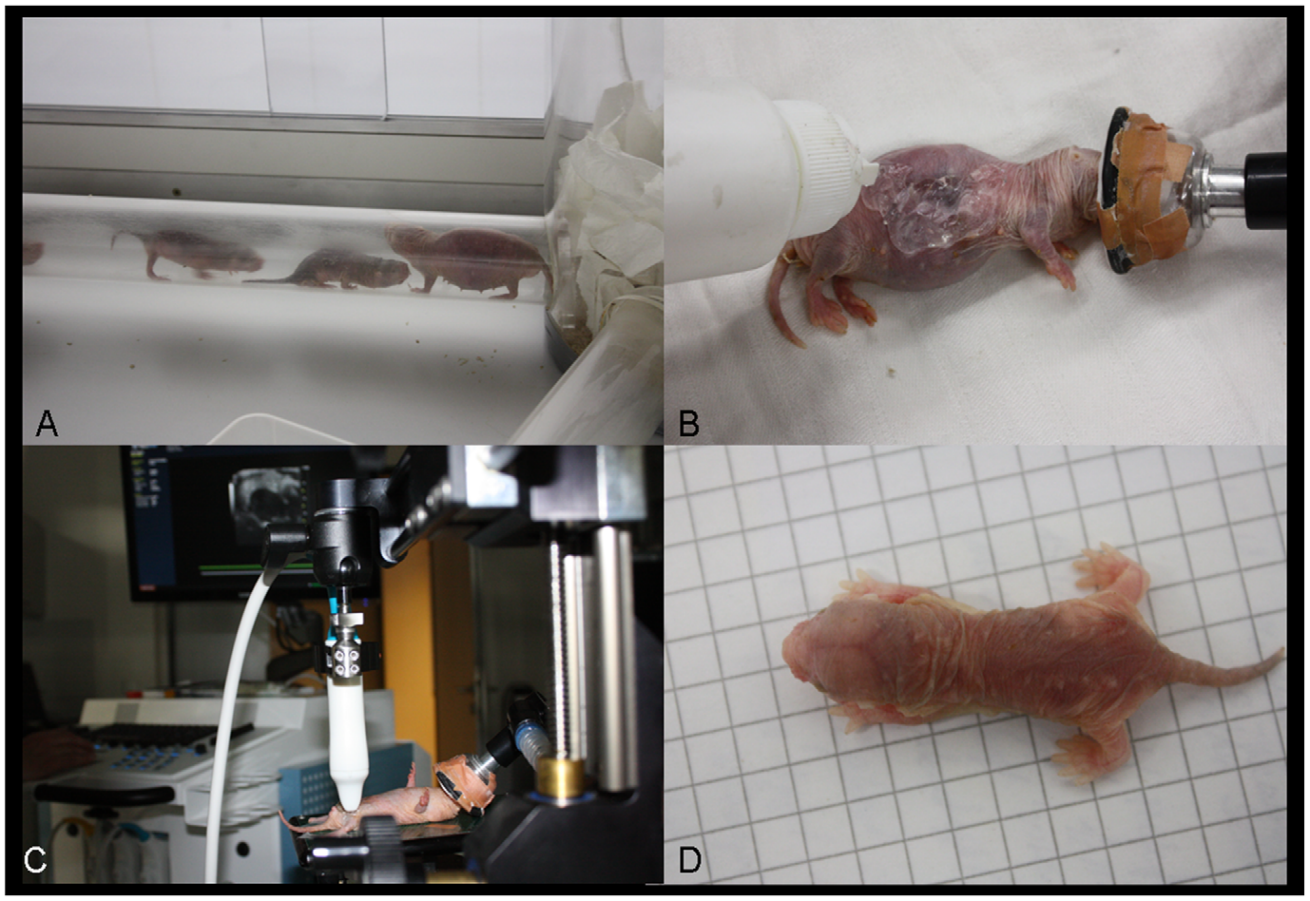
Captive management and examination of naked mole-rats. (A) Artificial burrow system of the captive naked mole-rat study colony. (B) Inhalation anaesthesia regularly used for immobilisation of naked mole-rats, maintaining. (C) Ultrasound in a naked mole-rat using the ultrasound biomicroscope. (D) Postnatal measurement of crown-rump-length.

The studied colony of naked mole-rats (*Heterocephalus glaber*, Rüppell 1842) was founded in September 2008 consisting of sixteen adult individuals, one queen (nulliparous before), seven females and eight males derived from the Albuquerque Zoo, New Mexico, USA. The colony was maintained at the Leibniz Institute for Zoo and Wildlife Research in Berlin in an artificial burrow system inside a large plexiglass box (2×1 m^2^) heated to 24° to 28°C with a constant high relative humidity of 40% to 70%. The burrow system consisted of a series of eight interconnecting clear acrylic plastic (plexiglass) tubes with a nest chamber, a toilet chamber, and a chamber where food was introduced, which was defined by the animals themselves (Fig. 1). The total tunnel length was about 5.70 m. The chambers contained wood bedding some twigs and unbleached paper tissue. The burrow system was cleaned on a daily basis. Fresh food was given daily ad libitum and included sweet potatoes, carrots, apples, bananas, beet root and cucumber. A cereal supplement containing vitamins and minerals was also provided once per week. All animals were checked on a regular basis for their reproductive status and habituated to the ultrasonographic examination procedure (see below).

After 11 month and two successful litters of one queen (Q1) the colony was divided into two. The queen (Q1) was left in the old system with all her recent offspring and three breeding males. A second colony was founded with two females and seven males that initially came with the colony. After seven successful pregnancies (P1 to P7) of two queens (Q1 and Q2) at present we harbour 63 animals.

Adult animals were identified via weight measurement. Newborn of the last five litters (L3 to L7) were marked by toe clipping as successfully performed previously [21], [22] according to a numerical pattern for identification. Sex ratio of the offspring was determined applying a technique of molecular sexing described by Katsushima *et al.* [23].

The IZW received the approval to keep and to breed naked mole-rats for research purposes only (#ZH 156; 23.09.2008) by the “State office of health and social welfare” (Landesamt für Gesundheit und Soziales, Berlin). All ultrasound examinations were performed during measures of routine health assessments and normal breeding management. All manipulations were performed in accordance with national and local laws/guidelines to conduce to optimization of husbandry and to establishment of a healthy breeding colony.

### Examination of pregnancies

The reproductive status of the potentially reproducing females was monitored on a regular basis. They were captured and put into a large plastic mask designed for inhalation of anaesthesia. Anaesthesia was achieved via the application of 5 Vol. % Isoflurane (Isoba, Essex) and oxygen with a flow rate of 2 l/min and maintained with a small mask (1.5 Vol. % Isoflurane in oxygen) (Fig. 1). As the ability of naked mole-rats to individually regulate their body temperature is limited [24], all procedures were performed on an electric heating pad, to prevent hypothermia of the animals. Because they are naked no further preparation of the animals was needed. Standard ultrasound gel was prewarmed in a hot water bath and used to increase coupling.

**Figure 2:**
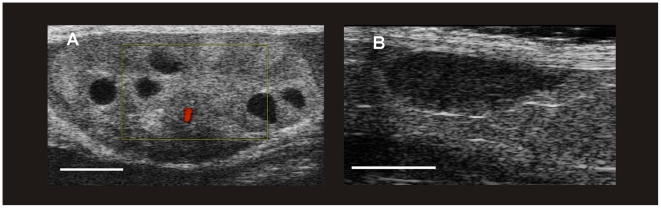
Ultrasonograms of the ovaries of naked mole-rats. (A) Active ovary with corpora lutea of a reproducing and pregnant queen. The colour-doppler-flow mode demonstrates the blood supply of an active corpus luteum of pregnancy. (B) Inactive ovary of a non-breeding female. (The white bar represents 2 mm.)

**Figure 3:**
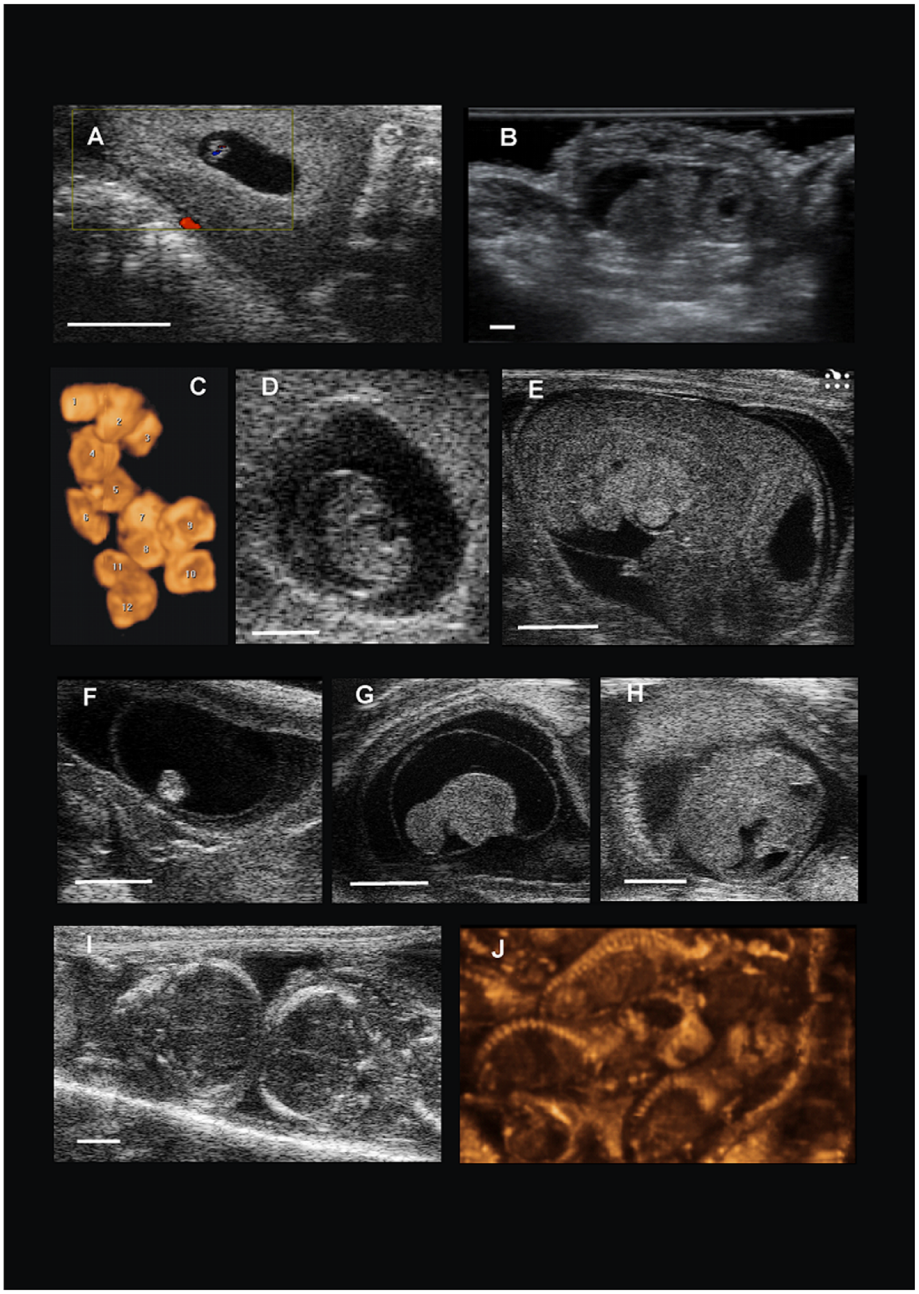
Ultrasonograms of different gestational stages in a naked mole-rat queen. (A,B) early embryonic vesicles at day 53 preP displayed with (A) a 75 MHz linear transducer (Vevo 2100, Visualsonics, Canada): a viable embryo can be detected using Doppler ultrasonography. (B) The same gestational stage displayed with a 5–14 MHz linear transducer (Voluson E8 Expert BT09, GE Ultraschall Deutschland GmbH, Germany). (C) 3D-reconstruction of embryonic vesicles in the reproductive tract of a pregnant queen (day 39 preP). (D) Early embryo at day 41 preP. (E) Longitudinal section of an embryo enveloped by its amnion at day 36 preP. Note the brain veswicles and the heart with the surrounding pericard. (F,G,H) Three stages of embryonic resorption again at day 36 preP. (I) Two fetuses next to each other at day 20 preP. (J) 3D reconstruction of close to term fetuses 2 days preP. (The white bar represents 2 mm.)

To track the individual pregnancies examinations were performed with two different high-resolution ultrasound machines. One was an ultrasound biomicroscope (UBM) with high-resolution linear transducers with frequencies up to 50 and 70 MHz (Vevo 2100, Visualsonics, Canada) which allowed a minimal resolution of about 20 µm. The second ultrasound unit had integrated options for 3D and 4D ultrasound (Voluson E8 Expert BT09, GE Ultraschall Deutschland GmbH, Germany). The 2D ultrasonograms presented (Fig. 2,3) are derived from the Vevo 2100 with the high-resolution transducers.

For all seven pregnancies (P1 to P7), the exact date of impregnation was not known as we did not perform systematic behavioural studies and mating activity was not observed. Therefore the gestational age is not presented as usual calculating the days *post conceptionem*. We here use the calculation of the time interval left until parturition, counting the days before parturition/*pre partum* (preP).

During examination measurements on embryonic structures were taken according to the gestational age, including the diameter of the embryonic vesicle (EV), the crown-rump-length of the embryo (CRL), the biparietal diameter (BPD) and the widest diameter of the thorax (TD). The measurements were taken as usually done in other mammal species to describe prenatal growth [25], [26]. Unusual intrauterine events and milestones in embryonic development were recorded and number of embryos was counted as exact as possible.

### Postnatal data collection

After parturition offspring were counted and weighed. We also measured the size of the newborn equivalent to the measured crown-rump-length and biparietal diameter *in utero* to determine an endpoint for the ultrasonographic growth curve. For this purpose the offspring were placed on a sheet of standardized squared paper and photographed (Fig. 1). Measurements were taken from a printed version of the picture and calculated relative to the scale which was also displayed on the picture. Depending on the status and stress level of the colony the number, weight and size of the offspring was checked on a regular basis.

### Data analysis

All statistical analyses were performed using SPSS ver. 18.0 (SPSS, Chicago, IL, USA). Values are presented as the mean ± standard deviation (SD). To establish a relationship between gestational age and morphological parameters BPD, EV and TD, linear regression was performed using the measurements in centimetres. For the sigmoidal CRL model a non-linear regression was performed using the logistic model by Peil [27] with estimated starting parameters. All other statistical tests are described in the text.

## Results

### Reproductive success

After founding a new captive colony in September 2008, six litters (L1 to L6) were delivered by one queen (Q1) within a time period of 23 month and one litter (L7) by a second queen (Q2). The first litter (L1) of Q1 was born 48 days after her arrival at our facilities. As pregnancy duration was reported to be about 70 days [9], [10] the nulliparous queen Q1 must have been pregnant prior arrival. To facilitate habituation to the new environment animals were not disturbed during the first weeks and hence the first pregnancy (P1) was not examined. The following pregnancies of both queens (P2 to P7) were monitored ultrasonographically on a regular basis. Breeding results are displayed in Table 1.

**Table 1:** Pregnancy data of the seven gestations.

No.	ID	Parturition			exams	res	em	mean birth	Parturition		1 month			3 month		
		real	predicted	interval				weight (g)	LS	sex	LS	%	sex	LS	%	sex
P1	Q1	06.11.2008		(48)	0				11		5	45		4	36	3,1
P2	Q1	16.05.2009		191	10	4	16		12		8	67		6	50	2,4
P3	Q1	06.12.2009	06.12.2009	204	5	1	12	1.73±0.16	11	4,7	8	73	2,6	8	73	2,6
P4	Q1	25.02.2010	24.02.2010	81	2	1	15	1.82±0.17	14	8,6	9	64	4,5	5	36	1,4
P5	Q1	24.05.2010	23.05.2010	88	1	3	12	2.03±0.27	9	7,2	7	78	5,2	7	78	5,2
P6	Q1	12.08.2010	16.08.2010	80	2	6	12	2.25±0.22	6	4,2	6	100	4,2			
P7	Q2	30.08.2010	30.08.2010	(130)	1	0	13	2.02±0.20	13	5,8						
Mean				128.8		2.3	13.3	1.93	10.9	28,25	7.2	71.2	15,15	6.0	54.5	13,17
SD				63.0		1.5	1.8	0.3	2.7		1.5	17.9		1.6	19.9	

*No.* = number of the respective pregnancies; *ID* of the respective queen; given is the *real* parturition date and the date which was *predicted* from the reference data from the preceding pregnancies, also the time *interval* between successive pregnancies (numbers in brackets are the interval after founding the colony); *exams* = number of ultrasonographic examinations per pregnancy; *res* = number of embryonic resorptions detected in ultrasonographic exams; *em* = initial minimal number of embryos before resorption; *LS* = litter size; *sex* = sexratio of the offspring; % = survival rate.

Queen Q1, did show long initial inter-birth intervals (191 and 204 days) which became shorter with successive pregnancies (81, 88 and 80 days). The mean interval was 128.8±63.0 days. The reproductive success in terms of litter size after parturition ranged from 6 to 14 (10.5±2.7 offspring, *N* = 6) (L1 to L6). The mean weight of the newborn was 1.90±0.27 g (L1 to L6) and significantly decreased with litter size (Spearman Rank Correlation, *r* = −0.477, *P* = 0.0018). The mean offspring survival rate was 71.2±17.9% (L1 to L6) after one month (weaning age) and 54.5±19.9% (L1 to L5) after three months. After this period none of the offspring died until present. Complete sex ratio of the offspring at parturition was determined only in L3 to L6 due to the fact that in these pregnancies parturition date was predicted (see below) and genetic samples could be taken from all the newborn. Sex ratio was slightly male biased at birth (0.58, L3 to L6) but female biased (0.43, L3 to L5) after three month of age.

Eleven months after the colony was founded, a second colony was established by separating two adult females and eight males from the original founder colony into a second enclosure. Within the next month the larger female, started developing external features typical for a queen such as an increase in the length of the vertebral column and gain of body mass. However, no full-term pregnancy developed. Regular ultrasound examinations were performed and two month after founding the second colony, early embryonic vesicles characteristic for 52 days preP were detected (see below). However, these embryos must have undergone resorption as in follow-up examinations no signs of pregnancy could be detected any longer. In the following month no signs of another pregnancy could be seen either, although the ovaries of this potential queen were enlarged and active similar to the ones of Q1 (see below). Instead, sonographic evidence of a subclinical endometritis was found five month later. The aetiology of this reproductive disorder is unclear. However, it could have been caused by reproductive failure due to embryonic resorption detected frequently in the individuals in this study (see below). Due to overpopulation in colony one and the unsuccessful reproduction in the second colony, three more adult females, of which one was already very large, and two males were shifted. After two weeks severe fighting for the queenship began in the second colony. Five individuals including the female which was the previous potential second queen and four males died or had to be euthanized due to severe injuries. One of the females was diagnosed to be in an advanced stage of pregnancy three months after introducing the new individuals into colony two. This new queen (Q2) delivered a litter of 13 offspring with a mean birth weight of 2.02±0.20 g. Including this litter the result of a significant correlation between litter size and birth weight is confirmed (L3 to L7, Spearman Rank Correlation, *r* = −0.317, *P* = 0.021).

### Ultrasonographic appearance of female reproductive organs

The ovaries of non-breeding females were hard to detect on a regular basis. They could be found close to the kidneys and were difficult to distinguish from the surrounding tissue (Fig. 2B). By ultrasound, the ovaries resembled an elliptoid structure. The mean diameter of the ovary, calculated from the means of the maximum length and width, was 1.84±0.42 mm (*N* = 14). There was no difference between the left and right ovary (Mann-Whitney-U-Test, *U* = 47.00, *P* = 0.913). The uterus of a non-breeding female was difficult to visualize due to its inactive status. The mean uterus diameter was 1.55±0.53 mm (*N* = 5). The kidneys were readily detectable, they appeared as a homogenous elliptoid structure in the ultrasound examination. Medulla and cortex could not be differentiated. The mean kidney diameter, calculated from the means of the maximum length and width, was of 5.86±0.81 mm (*N* = 16) which significantly differed from the mean diameter of the ovaries (Mann-Whitney-U-Test, *U* = 0.00, *P*<0.0001). This is pointed out to avoid misleading diagnoses confusing ovaries and kidneys in the ultrasound examination.

The ovaries of a pregnant female (Fig. 2A) were prominent and had a mean diameter of 4.54±1.28 mm (*N* = 7 measurements in one queen). This diameter was significantly larger than in non-breeding females (Mann-Whitney-U-Test, *U* = 0.00, *P*<0.0001). The difference in mean ovarian diameter in non-breeding females and the queen leads to a calculated volume difference of 1∶17. The mean diameter of the ovaries of the pregnant female significantly increased with gestational age (Spearman-Rank-Correlation, *r* = 1.00, *P* = 0.0028). Ovarian structures such as medulla, cortex and functional structures could be clearly distinguished. The corpora lutea were not easy to differentiate from the surrounding tissue but had an anechoic potentially fluid filled center. The mean diameter of these cavities was 0.73±0.08 mm (*N* = 6 at day 31 preP) (Fig. 2A). An exact counting of the corpora lutea to estimate the initial ovulation number was difficult and for obvious reasons it was not performed.

### Prenatal development

The prenatal development of the naked mole-rat offspring was followed sonographically. Detectable characteristics of embryonic structures were recorded using a 3D-ultrasound unit and an ultrasound biomicroscope (UBM). Using a linear transducer with the highest frequency (70 MHz) very small structures (20 µm) could be differentiated.

#### Embryonic development

On day 60 preP a pregnancy could not yet be confirmed but the endometrial layer was clearly distinguishable from the myometrium and some fluid was visible between the endometrial layers. It was not clear if there were already embryonic structures developed. Round anechoic structures with a diameter below one mm identified as embryonic vesicles could be first detected on day 53 preP. Initially in P3 this was believed to be a periimplantative stage when observed with the 3D-ultrasound unit. However, examination of the same stage in P6 with the more sensitive UBM revealed a viable embryo within the same anechoic round structures. The heartbeat could be detected using the Color-Doppler-Mode (Fig. 3A,B). Hence, we suggest that day 53 preP is a postimplantative stage.

Up to now there is too few data to exactly detect the period where implantation occurs especially as different ultrasound units give different detailed information. However, implantation is assumed to occur within the time period between 60 and 53 days preP. Generating a 3D view on the fluid filled structures in the uterus was especially useful to count the implantation sites (Fig. 3C, J).

On day 46 preP the viable embryos already had a body length of about two mm and were detected within their fluid filled embryonic cavities. Discoid shaped placental structures of around 3 mm in thickness were visible. On day 41 preP the brain vesicles with the hyperechoic *Plexus choroideus* were distinguishable which had a size of below one mm. The umbilical cord was detectable and the heart action could be characterized by pulsed-wave Doppler ultrasonography with the 3D-ultrasound unit. The heart-rate of the embryo was about 150 beats/min and lower than that of the mother with about 190 beats/min. There was still a lot of fluid inside the extra-embryonic compartments relative to the size of the embryo. Since the resolution of embryonic structures was highly dependent on frequencies and ultrasound units used, it is not easy to characterise general ultrasonographic milestones for embryonic development. A good marker was the shape of the embryo. At day 41 preP it was not much differentiated and “cigar shaped” (Fig. 3D). At day 36 preP the sagittal section had the appearance of “curled layers” (Fig. 3E). At day 32 preP the heart was already structured in four chambers, the pericardial sac could be distinguished, the stomach was filled with fluid and the ultrasonographic difference between the lung and the liver, the anatomical structure of the diaphragm, was detectable. The embryo now filled most of its embryonic cavity and was only surrounded by a small amount of fluid. The ratio of the length of the head to the rump was approximately 1∶1. There were no ossification centres detectable. The time period of embryogenesis in naked mole-rats is assumed to last approximately until day 30 preP. This is based on ultrasonographic morphological features when compared to data of other species where the point of transition from embryo to fetus is known [26].

#### Fetal development

At day 24 preP the size of the embryonic cavity was only as large as the fetus itself (Fig. 3I). Fetal structures like eyes, vertebral column and the limbs were clearly distinguishable. The fetal development is mainly characterised by fetal growth and changes in body proportions as well as spontaneous movement which are detectable by ultrasound. Depending on the sonographic technique used internal organs of the fetus could be displayed in detail. The uterus of the queen became very “crowded” and her body circumference increased rapidly in this last third of pregnancy. Around 10 days preP fetal growth rate seemed to slow down.

#### Embryonic resorption

Resorption of conceptuus affecting all embryonic stages were found in all five pregnancies examined in Q1 (P2 to P6, Fig. 3). The frequency of examinations was not high enough to follow the process of resorption in detail, but the detection of several resorption sites gave an impression of this process. Embryonic resorptions were detected in the interval of 53 to 29 days preP. Hence, the death of the conceptuus mainly happened within the time period of embryogenesis. In P3, one embryo in resorption had a length of about 1.5 mm on day 33 preP while its living siblings had a crown-rump-length (CRL) of more than 10 mm. In P5 three embryonic resorptions in different stages were detected on day 32 preP next to living and normally developed fetuses (Fig. 3E–H). In resorption one, the embryonic cavity was fluid filled and the arcuated embryo measured 2 mm. Head and rump of the embryo could not be distinguished. In resorption two, the embryo itself could not be detected, only a fluid filled cavity with an embryonic membrane. Resorption three was characterized by condensed placental tissue. The embryo itself could not be visualized either. After the embryo itself died embryonic fluid was resorbed first and at the end only unstructured tissue was seen within the uterus. Different to other species where embryonic resorptions were detected ultrasonographically [28], the resorption process itself seems to be very slow and the integrity of the embryonic cavities is still intact while the embryo is already deformed and nearly not detectable anymore. Altogether ten resorption sites were detected in six (P2 to P7) pregnancies which gives a minimum initial intrauterine litter size of 13.3±1.8 embryos (Table 1). It can not be evaluated if even earlier embryonic loss occurs and how many fertilizations or rather implantations occur in a naked mole-rat queen. No correlation between the frequency of examinations and the observed resorption events was seen.

### Mathematical models to describe prenatal growth

**Figure 4:**
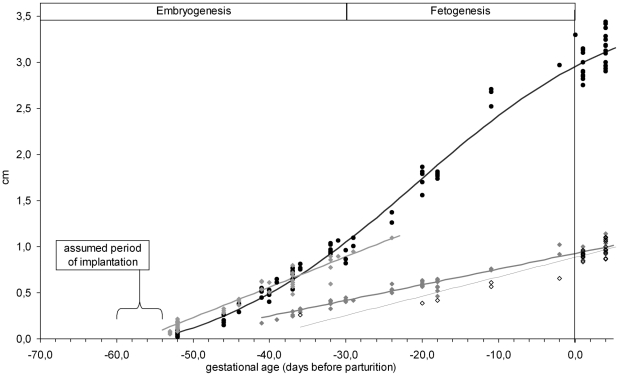
Prenatal growth in naked mole-rats. Biometric parameters (in cm) are displayed as a function of gestational age. Dots represent the data points and lines the regression curves (equations are given in the text). *Light grey* = mean diameter of the embryonic vesicle (EV), *black (thick)* = crown-rump-length (CRL), *dark grey* = biparietal diameter, *black (thin)* = widest diameter of the thorax.

Pregnancy was first ultrasonographically detected in P2 of Q1 by visualizing embryonic vesicles with living embryos (indicated by heartbeat) on day 46 preP. At this stage the embryos itself had a length of 1.9±0.4 mm (*N* = 5). Ultrasonographic examinations were performed ten times over the course of P2. Morphological parameters such as diameter of the embryonic vesicle (EV), crown-rump-length (CRL), biparietal diameter (BPD), and thoracic diameter (TD) were measured. Examination length was restricted to reduce the duration of the anaesthesia therefore not all measurements were taken on all conceptuus. Furthermore, with advancing gestation, the conceptuus overlapped each other, hindering the display of all parameters. Measurements were plotted against gestational age calculated backwards from the day of parturition in a pregnancy graph. Using the pregnancy graph from P2 gestational age of the next four pregnancies of Q1 (P3 to P6) was calculated after the first two examinations. Pregnancy in P3 was confirmed on day 53 preP when embryonic vesicles could be detected. The second exam was performed on day 41 preP displaying life embryos with a mean CRL of 5.1±0.5 mm (*N* = 4). Due to a much shorter inter-birth interval than observed between the previous pregnancies, P4 was diagnosed not earlier than on day 36 preP and confirmed in a second ultrasound exam on day 20 preP. In P5, pregnancy was diagnosed in mid-gestation at day 32 preP but not examined anymore due to conflicts in the second colony. In P6, two examinations on days 52 and 31 preP were performed. By comparing the data to the pregnancy graph derived from measurements of P2 (and the other previous pregnancies), expected parturition dates were predicted for P3 to P6. In P3, parturition occurred on the exact predicted date, in P4 and P5 one day after the predicted date and in P6 four days before the predicted date. In Q2, the one pregnancy (P7) was diagnosed on day 35 preP. Examination was not repeated due to the conflicts in this colony. However, delivery in Q2 occurred exactly on the predicted parturition date calculated from the gestational data from Q1. Data points measured for all the pregnancies are displayed in Fig. 4. Since the course of gestation was similar in all pregnancies and the date of parturition did not vary much, all data points were pooled and used for regression analysis to model prenatal growth. For CRL a logistic model [27] was used. A non-linear regression was performed calculating the gestational age (age) as a function of CRL. For BPD, EV and TD a linear model was used to calculate the gestational age (age) as a function of these parameters. The measurements of the biometric parameters (CRL, BPD, EV and TD) were used in centimetres (cm) for the calculation of the growth models and should therefore also be given in cm when using the models to predict gestational age. As the gestational age is calculated in days *pre partum* the resulting value will be a negative number.

- age = −14.08 * ln ( ( 4.03 / (CRL+0.33) )−1)−20.70 R^2^ = 0.984
- age = 55.77 * BPD−52.21 R^2^ = 0.969 F(1.69) = 2157.35 p<0.0001
- age = 28.26 * EV−55.72 R^2^ = 0.942 F(1.44) = 709.74 p<0.0001
- age = 80.62 * TD−56.27 R^2^ = 0.969 F(1.29) = 199.827 p<0.0001

As the first pregnancy was confirmed on day 53 preP, pregnancy duration was at least 53 days long.

## Discussion

The data presented here gives an insight in the prenatal development of the eusocial naked mole-rat. We produced reference data for the evaluation of gestation in this species using data of seven pregnancies in two individuals The exact prediction of the parturition date for Q2 using ultrasonographic data from Q1 confirms that our results can successfully be used as reference data for gestation in naked mole-rats. In future studies this data needs to be extended with data from pregnancies of more individuals. However, detailed data evaluation on reproductive success illustrates that the two queens performed “average” pregnancies and hence the gestational data will be suitable for other individuals. The mean litter size in our colony (10.9±2.7 offspring, *N* = 7) did not differ from data collected in other captive colonies (12.3±5.7 offspring, *N* = 84 litters in 16 females) (unpaired t-test with Welch correction, *t* = 1.171, *df* = 11, *P* = 0.266) (Jarvis, 1991). Overall mean birth weight (1.93±0.25 g, *N* = 53) did not differ from data observed in a colony of wild caught individuals (1.86±0.33 g, *N* = 155 offspring (14 litters, 5 females)) (unpaired t-test with Welch correction, *t* = 1.614, *df* = 118, *P* = 0.109) [11]. Weaning success in other colonies was found to be variable (14 to 52%) and there was a strong indication that rapid colony growth contributes to high pup mortality [9]. Our pup survival rates were within this range. In our colonies postnatal mortality after one month seemed to be correlated to actual size of the breeding colony because of the limited available space. Although actual mating was not observed in our colony we predict that mating occurred 8 to 11 days postpartum (for the latter pregnancies) as observed by others [10]. In non-pregnant but reproductively active females regular oscillations (40 days) of urinary progesterone levels occur and progesterone levels may reach high levels over a prolonged period (up to 80 days) [15]. This together with an unsuccessful mating might explain the long parturition intervals of 191 and 204 days seen for initial pregancies.

We could document for the first time that embryonic resorptions occur in this species. This might have led to the infertility observed for the potential queen of the second colony. Furthermore resorption might be a way of regulating offspring size and a higher prenatal resorption rate might increase postnatal survival rate as seen in P6.

Without ultrasonographic examination the external signs of a pregnancy are visually evident on day 40 after mating [9], corresponding to a gestational age of 30 days preP at which embryonic development is almost completed. A similar gestational stage was diagnosed by Hildebrandt *et al.* [29] using the technique of ultrasound and a 7.5 MHz-transducer in naked mole-rats for the first time. In their study the detected conceptuus were about 0.8 cm in length and the gestational age was estimated to be 40 days of pregnancy [29]. Using the growth curves (equation I) calculated in this study an 0.8 cm sized conceptus (assuming adequate to CRL) would have a gestational age of 33.8 days preP. This validates the usage of the here developed growth curves for estimating gestational age in other naked mole-rat queens. Furthermore by using high-resolution ultrasound and UBM a pregnancy diagnosis in naked mole-rat queens can now be performed up to three weeks earlier then previously, around day 50 preP. It gets apparent from our study, that different ultrasound techniques will aid the detection of more details within distinct gestational ages. From the measured biometric parameters the examined developmental state can be calculated exactly and parturition date can be predicted easily. The UBM technique gives very detailed insight into prenatal processes but the gross overview on the uterus as a whole gets sometimes lost. Instead the 3D-technique is suitable to count implantation sites and helps to keep the overview. Therefore the use of different ultrasound technique was advantageous to generate information on the gestation in such a delicate species.

According to the long gestation, 70 days, prenatal development in naked mole-rats is very slow. Preimplantation period is prolonged compared to other rodents of this size [30]. Implantation occurs at least ten days after fertilization in the interval between 60 and 53 days preP. In addition an asymptotic growth phase is observed in the period before parturition which is very unusual for altricial mammals.

The question arises whether the mode of parental care in this species is altricial or rather precocial as the usual criteria for altricials being born “blind and naked” is obviously difficult to apply in this species. As the initial definition of altriciality and precociality was applied to birds, Martin [31] provided a list of the main characteristics of the two reproductive strategies in mammals. The altricial type is usually characterised by (I) small body size and living in nests, (II) naked born offspring with eyes and ears closed, exhibiting imperfect homeothermy, (III) lower jaw is incompletely developed and teeth erupt quite lately, (IV) gestation period is relatively short and litter size and teat count are large, (V) low mobility of infants at birth, (VI) relative small brain size of neonate and adults, (VII) nocturnal habits of adults. Some of the criteria are fulfilled by the naked mole-rats and some are not. Naked mole-rats are small and live in nests. At birth the eyelids are fused and incisors have not erupted, eyes open 30 days after birth [9]. Naked mole-rats exhibit imperfect homeothermy throughout their lives [24]. The long gestation contradicts the large litter size and teat count. Within a few hours of birth pups are able to walk. It is not quite clear whether the brain is more developed at birth compared to other rodents which could also be a reason for the long gestation period.

The slow prenatal development raises the question why the naked mole-rat has such a long gestation period relative to its size. We assume that the answer to this question can be found in the social structure of this species. A naked mole-rat queen delivers a large litter (10.9 offspring) after an average gestation length of 70 days. According to theoretical models, that correlate length of gestation to body size the predicted pregnancy duration of a naked mole-rat would be about 30 days, delivering a mean number of 5.5 offspring [13], [32]. If this was the case the same number of offspring could be born in the same amount of time. However, this would mean that by the time the second litter was born, the previous litter might not be weaned yet and would still be dependent on the care of the queen. The exceptional long pregnancy of the naked mole-rat could assure that the preceding offspring is fully independent of the queen and can actively participate in the rearing of the next litter (Braude, pers. comm.). Interestingly litter size is twice as large as for other rodents of similar size. This mechanism seems to ensure that total number of offspring is not compromised by the long pregnancy duration which is also about twice as long compared to other rodents of similar size. This theory is strengthen by the observation that naked mole-rats can give birth and successfully nurse almost twice as much offspring as there are mammary glands on the queen, which is in strong contrast to other mammals [17]. It is tempting to postulate that the long pregnancy enables the queen to produce such large numbers of offspring within one litter and hence might be an adaptation to the social structure of the naked mole-rat colony. However, long gestation periods and slow prenatal development is also observed in solitary bathyergid species [33] and therefore these traits may not solely be linked to the social organization of naked mole-rats. In the phylogentic tree of bathyergid species the naked mole-rat is at the base [34], [35] assuming that these reproductive and developmental traits might be an ancestral characteristic [33].

In addition, the naked mole-rat exhibits exceptionally longevity when compared to its body size [36], making this rodent an interesting model for research into aging. In both mammals and birds slow embryonic development, manifested by a low acceleration of embryonic growth rate, was shown to be an adaptation to delay aging [37] and rate of aging in mammals is correlated to postnatal growth rate [38]. Hence, the long developmental period in the naked mole-rat might support its exceptionally long life span. In future it will be interesting to elucidate the underlying molecular mechanisms for this process.

In the remaining section we will discuss the reproductive biology of the naked mole-rat in the light of the life-history-theory [39]. In stable populations the life time reproductive effort (LRE) was found to be a constant and a female can produce a mass of offspring approximately equal to 1.4 times her own body mass [40]. Charnov, Warne & Moses [40] estimated the LRE from life history parameters of several species by applying the following equation: LRE = (((litters/year)*litter size*average adult life span*offspring mass at independence)/adult mass at first reproduction). For Rodentia the LRE varied from 1.03 to 2.16. We examined the reproductive biology of the naked mole-rat within the theoretical concept of the constant LRE. We used data by Jarvis and Sherman [20] which gives the following mean values: 4.5 litters per year, 11.3 offspring per litter, 11 g at weaning age, 40 g mean adult weight and weight of the female at first reproduction. Since the time of maturity in naked mole-rats varies between 7.5 month and >16 years, dependenting on the opportunity of becoming a breeding female, it is hard to estimate the average adult life span, the time from maturity to death of the individual. Therefore mean longevity is assumed to be 10 years for all naked mole-rats and a maximum of 23 years for a breeding female. Assuming a mean reproductive period of 10 years for the queen the calculation of the LRE for a naked mole-rat queen would give the following LRE = ((4.5 litters/year * 11.3 offspring * 11 g * 10 years)/40 g) = 139.8. This would mean that a naked mole-rat queen can produce a mass of offspring of approximately 140 times her own body mass. Even if parameters are adjusted and reproductive periods are shortened, the LRE of the queen naked mole-rat will be at least an order of magnitude larger than the theoretical LRE of about 1.4 for other mammals. The colony size of wild naked mole-rat colonies was observed to be as large as 300 individuals with a mean of about 80 [20]. Assuming a colony size of about 100 individuals, including 50 females, calculated LRE would be 2.8 (140 devided by 50). This value falls within the range of the LREs calculated for other rodents (1.03 to 2.16). This demonstrates that the naked mole-rat queen produces a mass of offspring equivalent to the mass of offspring that would be produced if all of the females would be reproducing. This fact fits well within the concept of an eusocial behavioural pattern as observed in insects [3]. Only in an eusocial environment it is possible that a single queen can devote that much energy for reproduction.
